# One-year retention of gait speed improvement in stroke survivors after treatment with a wearable home-use gait device

**DOI:** 10.3389/fneur.2023.1089083

**Published:** 2024-01-11

**Authors:** Brianne Darcy, Lauren Rashford, Nancey T. Tsai, David Huizenga, Kyle B. Reed, Stacy J. M. Bamberg

**Affiliations:** ^1^Moterum Technologies, Inc., Salt Lake City, UT, United States; ^2^Department of Mechanical Engineering, University of South Florida, Tampa, FL, United States

**Keywords:** gait device, gait speed, iStride, stroke rehabilitation, walking speed, retention

## Abstract

**Background:**

Gait impairments after stroke are associated with numerous physical and psychological consequences. Treatment with the iStride^®^ gait device has been shown to facilitate improvements to gait function, including gait speed, for chronic stroke survivors with hemiparesis. This study examines the long-term gait speed changes up to 12 months after treatment with the gait device.

**Methods:**

Eighteen individuals at least one-year post-stroke completed a target of 12, 30-minute treatment sessions with the gait device in their home environment. Gait speed was measured at baseline and five follow-up sessions after the treatment period: one  week, one  month, three months, six months, and 12 months. Gait speed changes were analyzed using repeated-measures ANOVA from baseline to each follow-up time frame. Additional analysis included comparison to the minimal clinically important difference (MCID), evaluation of gait speed classification changes, and review of subjective questionnaires.

**Results:**

Participants retained an average gait speed improvement >0.21 m/s compared to baseline at all post-treatment time frames. Additionally, 94% of participants improved their gait speed beyond the MCID during one or more post-treatment measurements, and 88% subjectively reported a gait speed improvement.

**Conclusion:**

Treatment with the gait device may result in meaningful, long-term gait speed improvement for chronic stroke survivors with hemiparetic gait impairments.

**Clinical trial registration:**

https://clinicaltrials.gov/ct2/show/NCT03649217, identifier NCT03649217.

## 1 Introduction

Over seven million stroke survivors currently live in the United States (1), and early projections indicate this figure may rise to over 10 million in the next decade (1–3). Improvements in medical interventions have reduced mortality (4, 5), however the disability after stroke is often long-term and remains an economic burden globally (1) and a personal affliction for well over 60% of stroke survivors (6–8) and their caregivers (9). Impairments after stroke can be widespread with effects to multiple physiologic systems. However, when questioned on rehabilitation goals, a majority of stroke survivors cite improving gait as a top priority (10, 11). Gait dysfunction is experienced by more than 80% of stroke survivors (12), and approximately 30–40% of stroke survivors have limited to no walking ability, even after completion of traditional rehabilitation (13, 14).

The motivation to improve gait is multi-faceted. Impaired gait is inefficient, with a metabolic cost 40–50% higher than neurologically intact individuals (15), which can lead to difficulties performing daily activities (16). Impaired gait contributes to abnormal joint loading, which can lead to musculoskeletal complications and pain (17, 18). Additionally, impaired gait is associated with an increased risk of falls (12, 19) which compromises safety and contributes to the seven-fold higher risk of fractures seen in individuals with a history of stroke (20). Adding to the potential physical consequences of falls, fear of falling can further limit community participation, which contributes to mental health issues related to isolation, among other things (21). The need to enhance gait training outcomes post-stroke is critical; however, access to effective, long-term treatment can be insufficient, especially for chronic stroke survivors with limited access to clinical environments and/or resources.

The assessment of gait function provides unique insight not only into the disability status and rehabilitation needs of stroke survivors, but their overall health as well. The most studied and utilized measurement of gait function is the measurement of self-paced gait speed (7, 22). Despite its simplicity, the utility of gait speed measurement is heavily emphasized in medical literature with its clinical value stated to rival routinely measured vital signs such as blood pressure and pulse (23). While commonly associated with quality of life, general health status, and functional abilities, gait speed measurement has additionally been praised for its predictive value with factors such as mortality (24), falls (25), and community participation (26, 27). Its versatility enables utilization within multiple settings, including home environments (28), earning gait speed the highest level of outcome measure recommendation by an expert chronic stroke panel (StrokEDGE) (29) and designation as a core outcome measure for chronic stroke within clinical practice guidelines published by the Journal of Neurologic Physical Therapy (30).

The iStride® gait device (31) (Moterum Technologies, Inc.) is a wearable gait treatment device designed for individuals with hemiparetic gait impairments caused by stroke (32, 33). The device, worn on the foot of the non-paretic limb during overground ambulation, features four kinetic wheels (34) which alter interlimb coordination.

**Figure 1:**
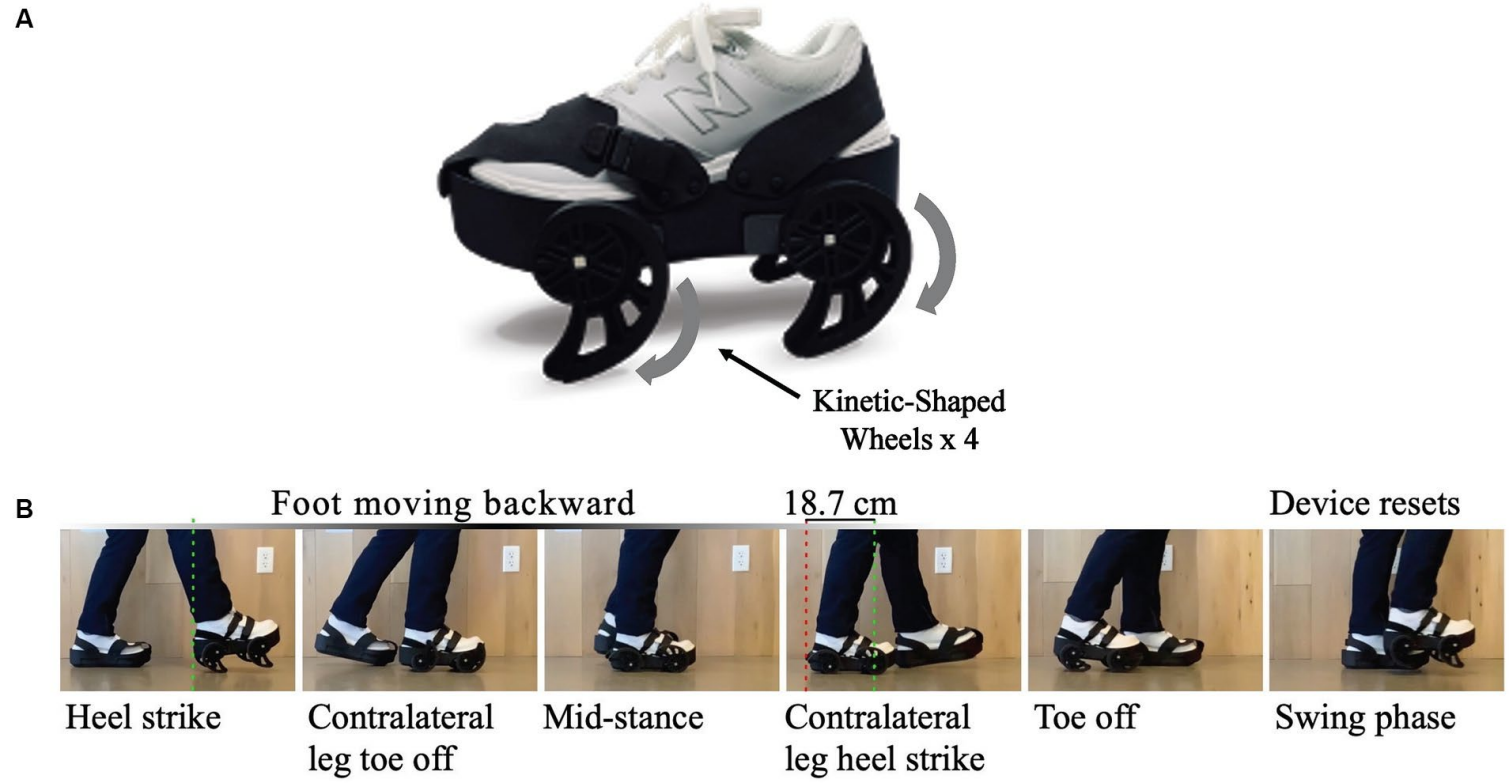
**(A)** The iStride^®^ gait device. **(B)** Gait device motion: As the user takes a step, the device pushes the nonparetic foot backward during stance. This exaggeration of the step length asymmetry for some users yields a more symmetric gait pattern once the device is removed and the user returns to overground walking without the device. In addition, the device encourages usage of the paretic leg by slightly destabilizing the nonparetic leg. A similar height but stationary platform is worn on the foot of the paretic limb for symmetry.

While ambulating with the gait device, the wheel motion causes a posterior translation of the non-paretic limb during mid-stance. This motion lengthens the steps on the paretic side – a mechanism which can reduce asymmetry for some individuals through error augmentation, as seen with split-belt treadmill training (35, 36). Additionally, the device creates a subtle destabilization of the non-paretic limb, which encourages greater usage of the paretic limb during the gait cycle and prompts the user to adapt to such instability throughout the treatment session and with repeated sessions. Encouraging usage of the paretic limb, a central principle of constraint-induced movement therapy for the lower extremity (37), is a key reason the device is donned to the foot of the non-paretic limb. Before-and-after studies (38, 39) conducted with 27 ambulatory post-stroke participants with heterogenous gait patterns have revealed post-treatment benefits to symmetry, functional mobility and balance, and gait speed after four weeks of treatment. We suspect that the device’s therapeutic effects combine uniquely to benefit each user individually. The gait device and its associated motion are shown in Figure 1. Further details of the device’s development, design, and mechanism can be reviewed in previously published manuscripts (32, 33, 36, 38).

The gait device was designed to be lightweight and portable, therefore, offering the potential for gait treatment to occur outside of clinical environments, a likely benefit for individuals with mobility restriction and difficulty accessing clinical settings. To investigate the feasibility and efficacy of treatment with the gait device in natural settings, a recent study explored treatment with the gait device in participants’ homes (39). Outcome measures centering on the functional aspects of gait that could be routinely measured in home settings were selected, including gait speed. Results after 12 treatment sessions revealed clinically relevant improvements, beyond the minimal detectable change or minimal clinically important difference, for both gait speed and functional balance, indicating an immediate post-treatment benefit for the home-use translation of this device (39). Retention of these gait improvements, however, remains undetermined, making the relevant, long-term clinical value unknown.

The objective of this study was to explore the long-term effects of treatment with the gait device in the home environment for individuals with gait impairments from chronic stroke. Specifically, this study investigates retention of the post-treatment therapeutic effect observed for gait speed by evaluating several follow-up time frames after the treatment period. Our hypothesis is that the gait speed improvement attained after gait device treatment will be sustained through all measured time frames.

## 2 Materials and methods

### 2.1 Selection criteria

Inclusion and exclusion criteria were identified as follows. Inclusion criteria: (1) age 21–80, (2) one or more cerebral strokes (all on the same side), (3) stroke occurred at least six months previously, (4) gait asymmetry (assessed by visual observation) but can walk independently with or without a cane, (5) no evidence of severe cognitive impairment that would interfere with understanding instructions, (6) not currently receiving physical therapy, (7) no evidence of one-sided neglect affecting ambulation, (8) adequate walking space within the home, and (9) weight less than 250 pounds. Exclusion Criteria: (1) uncontrolled seizures, (2) pregnancy, (3) metal implants (stents), (4) chronic obstructive pulmonary disease, (5) uncontrolled high blood pressure, (6) myocardial infarction within the last 180 days, (7) head injury within the last 180 days, or (8) a history of a neurologic disorder other than stroke. Additionally, during the period of treatment with the gait device, participants were excluded if the supervising physical therapist observed concerns regarding the participant’s ability to complete the treatment safely. Recruitment occurred during the months of July 2018 through September 2018. Treatment occurred between July 2018 and December 2018. All study-related follow-up was completed in December 2019.

Eighteen individuals with chronic stroke participated in this study, which features the long-term gait speed follow-up results from our home-based study with the gait device (39). This prior study reported results from a sample size of 21 participants. We derived the sample size for this study using power analyses from two previous studies using the gait device (32, 38). In the first study (32), the *t*-test was powered between pre-treatment and post-treatment data in healthy individuals and calculated an effect size of 0.68 for step length difference, resulting in an estimated minimal sample size of 18 participants. We initially included a higher number of participants since we expected more variation when testing on individuals with stroke. The second study (38), based on a pilot in-clinic study using the device with individuals with stroke, calculated an effect size of 0.71 for gait speed. A power analysis based on gait speed showed that statistical findings from 21 participants would obtain a power of 0.85. This power analysis does exclude one participant who started at a very fast walking speed of 1.14 m/s (and ended with a speed of 1.45 m/s), which is uncommonly fast for an individual with stroke; all of our participants in this study started with a gait speed less than 1.0 m/s. Note that these studies used step length asymmetry as a primary measure (which is not a variable in this study). Between the one-week and 12-month follow-up sessions, three out of the 21 participants did not complete all follow-up sessions. Since we are reporting repeated-measures statistical tests in this study, only the results of the 18 participants who completed all outcome assessments at all time periods will be included.

### 2.2 Experimental setup

The study followed a single group, before-after design with multiple follow-ups. Eligibility verification included an initial phone screen followed by a home visit to confirm compliance with eligibility criteria and to assess the home environment for suitability of device treatment. After consenting to participate, the participants’ gait parameters were measured at baseline (approximately one week before starting treatment), followed by four weeks of treatment with the gait device. After treatment was complete, gait speed was measured at five follow-up time frames: one week, one month, three months, six months, and 12 months post-treatment. At the final follow-up session, participants were provided a questionnaire regarding their clinical trial experience and observed gait changes after treatment with the gait device. All study aspects were performed within the participants’ home environments and were overseen by licensed, non-employee physical therapists hired as contractors for clinical trial data collection. This study was approved by the Western Institutional Review Board (IRB) and was confirmed to meet ethical standards for research with human participants. The study was registered with the identifier NCT03649217. Each participant signed a consent form that was approved by the Western Institutional Review Board prior to their study inclusion.

### 2.3 Treatment sessions

The participants were treated using the gait device in their home environment three times per week for four weeks (for a target of 12 treatment sessions). During each treatment session, the participant wore the device on their non-paretic foot. An approximate height-matched platform was worn on the paretic foot. The participants ambulated over ground on the gait device in their home environment for a maximum of 30 minutes during each treatment session. Rest breaks were provided at five-minute intervals (or more frequently if requested by the participants). Ambulation on the device was supervised by licensed physical therapists who provided the level of mobility assistance needed for participant safety and comfort while ambulating on the device. No other treatment or physical therapy services were provided to the participants during the treatment period.

### 2.4 Gait assessments

**Figure 2:**
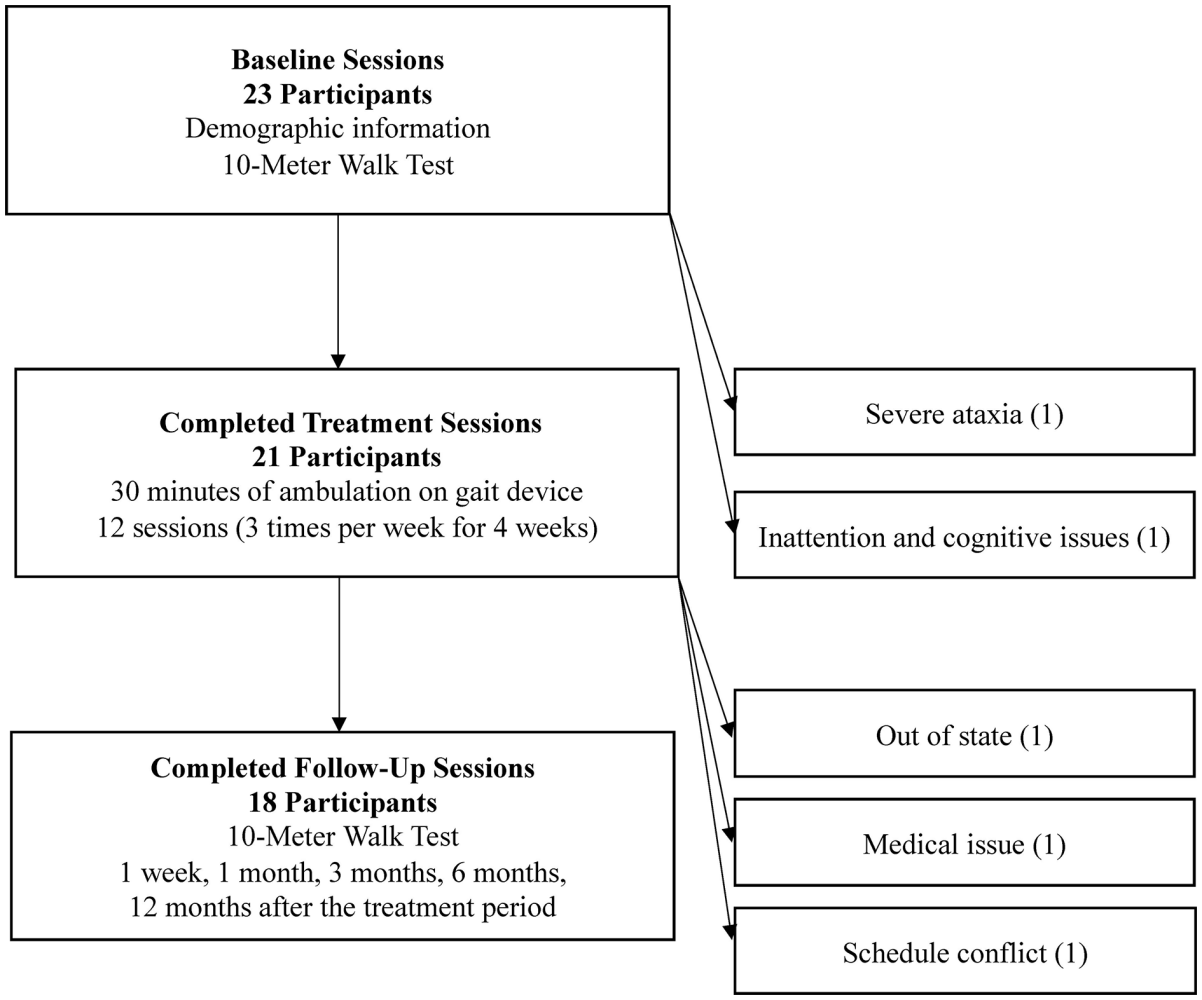
Study participants at each stage. Key study activities are listed within each phase, and reasons for non-participation are available to the right of each phase heading.

Gait speed assessment occurred at baseline (approximately one week before treatment) and at five follow-up time frames: one week, one month, three months, six months, and 12 months after the four-week treatment period. Gait speed was measured using the 10-Meter Walk Test (10MWT) at their comfortable walking speed. The protocol specified a 12-meter course using a measured distance of 10 meters and an untimed acceleration/deceleration distance of 1 meter. Three trials were conducted and averaged to determine the participant’s gait speed. For each participant, the most ideal home location (both for treatment with the gait device and for gait speed assessment) was identified by the therapist. This determination was based on identifying the longest straight path for walking in the home that was without thresholds, obstacles, or other walking surface changes. Testing setups were kept consistent within each participant’s environment and across all time frames to allow for within-subject comparisons. Figure 2 shows the study procedures and timeline.

### 2.5 Data analysis

Normality of data was checked using the Shapiro–Wilk test. Sphericity was evaluated using Mauchly’s test for sphericity, and corrections using Greenhouse–Geisser estimates were applied if sphericity had been violated. A one-way repeated measures ANOVA test was conducted with gait speed as the dependent variable and time frame as the independent variable (baseline and five follow-up time frames). When statistical significance was found (using an alpha of 0.05), Tukey’s honestly significant difference post-hoc test was performed with Bonferroni corrections. Statistical analyses were performed using SPSS Version 26 software (IBM Corporation, Armonk, NY).

To monitor for the meaningfulness of gait speed changes, we compared each individual’s gait speed change as well as the study group average to the minimal clinically important difference (MCID) for gait speed improvement (40). The MCID refers to the smallest amount of change in an outcome that might be considered “important” to the patient or clinician. Additionally, the speed of an individual’s gait also corresponds with their ability to participate within the community. A study using walking data and activity monitors by Fulk reported that a comfortable gait speed of 0.49 m/s discriminated between household and community ambulators and a speed of 0.93 m/s discriminated between limited and unlimited community ambulators (27). To monitor for changes in expected community participation ability, each participant’s walking speed was compared to these gait speed classifications, as well as a “normal walking speed” classification (>1.2 m/s) as characterized by Fritz and Lusardi (23), during each study time frame. Finally, participant responses to a questionnaire regarding clinical trial experience and subjective gait observations after treatment were manually tabulated for the percentage of positive or negative responses to each questionnaire item.

## 3 Results

### 3.1 Participants

Twenty-three participants were initially included for study participation. Figure 2 shows the study activities and the total number of participants after each study phase. Key study activities are listed within each phase, and reasons for non-participation are available to the right of each phase heading. The discussed results and analysis center on the 18 study participants that completed all assessments through the twelve-month follow-up.

The demographic characteristics of the 18 study participants are shown in Table 1.

**Table 1:** Participant demographics.

ID	Sex	Weight (kg)	Age (years)	Time since stroke (months)	Side of hemiparesis	AFO?	Assistive device during 10MWT?	Minutes of treatment
A	M	73	53	24	Left	No	No	295
B	F	82	77	15	Left	No	Cane	170
C	F	91	44	14	Left	No	No	270
D	M	86	63	53	Left	Yes	No	285
E	F	86	47	28	Right	Partial^a^	No	215
F	F	101	69	89	Left	No	No	330
G	F	61	46	308	Left	No	No	360
H	F	83	50	80	Left	Yes	No	210
I	M	100	61	22	Left	Yes	No	290
J	M	109	51	50	Left	Partial^a^	No	360
K	M	85	62	28	Left	No	No	295
L	F	113	58	92	Left	No	No	360
M	F	68	54	21	Right	No	No	280
N	M	107	52	130	Right	Yes	No	360
O	M	79	64	30	Right	Yes	No	260
P	M	90	55	13	Right	Yes	SBQC	355
Q	F	78	53	32	Left	No	SBQC	360
R	M	61	61	46	Left	Partial^a^	No	165
	9 Male 9 Female	Mean 86 (SD 15)	Mean 57 (SD 8)	Mean 60 (SD 70)	5 Right 13 Left	6 Yes 9 No 3 Partial	15 No 1 Cane 2 SBQC	Mean 290 (SD 66)

a Partial: participants who used an AFO occasionally at baseline, but did not use during the study.

SBQC, small base quad cane.

### 3.2 Statistical findings

**Figure 3:**
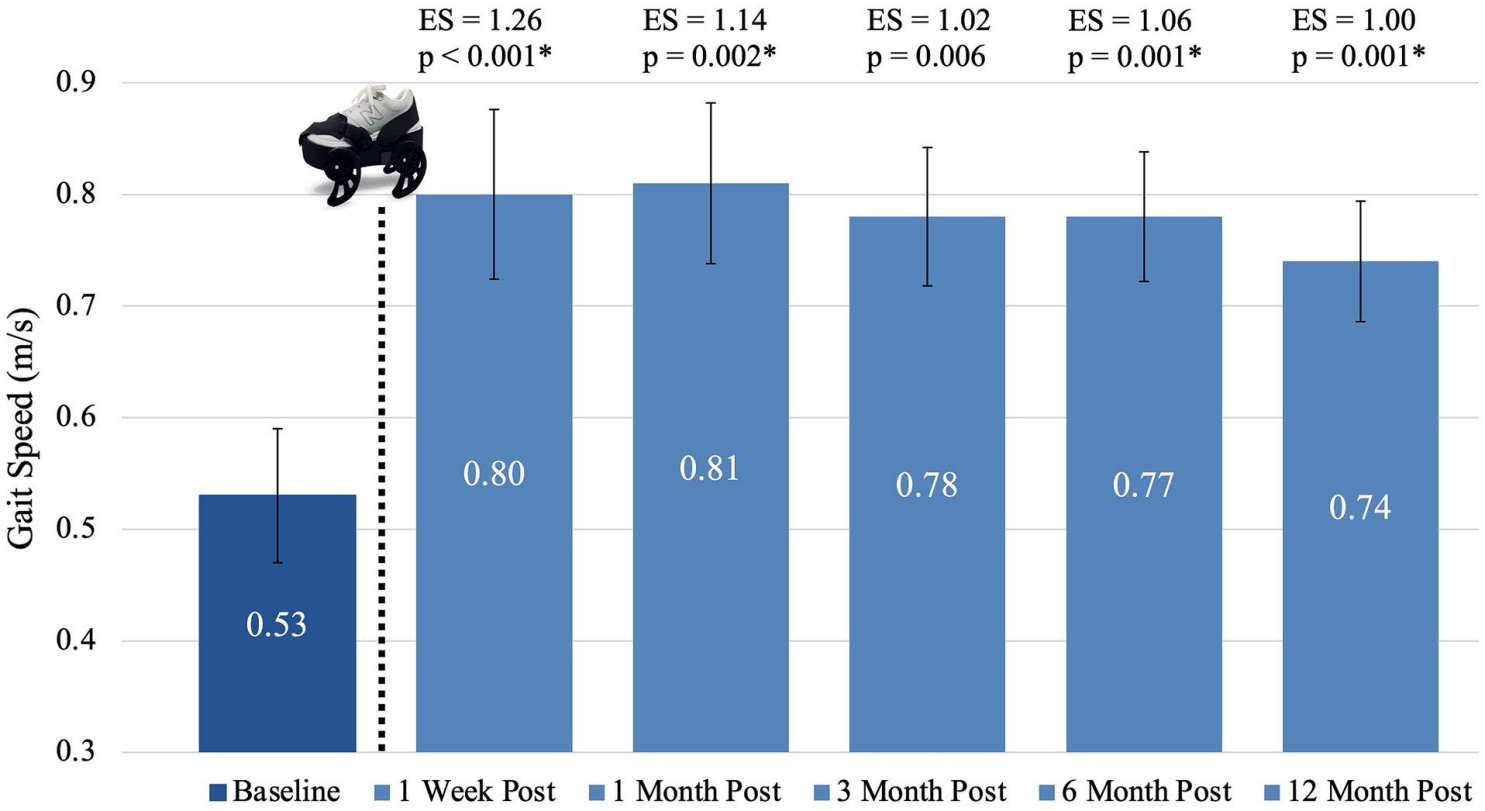
Mean gait speed at each time frame. The dotted line and gait device image represent the sequence within the study activities that the device treatment occurred. An asterisk (*) indicates statistical significance (*p* < 0.0033) compared to baseline. Bars represent standard error. ES, effect size.

Data for each time period followed a normal distribution (*p* > 0.2 on the Shapiro–Wilk test for all periods). Sphericity was violated (*p* < 0.05 on Mauchly’s Test of Sphericity), so Greenhouse–Geisser corrections were applied. Repeated-measures ANOVA revealed statistical significance for gait speed (measured using the 10MWT at comfortable gait speed) after treatment with the gait device {*F*(2.871, 48.815) = 9.195, *p* < 0.001}. Post hoc analysis revealed statistically significant differences from baseline to all follow-up time frames except three month (*p* < 0.0033, the Bonferroni corrected alpha based on 15 observations). Comfortable walking speed increased 0.27 m/s (*p* < 0.001) from baseline to one week post-treatment, 0.28 m/s (*p* = 0.002) from baseline to one month post-treatment, 0.25 m/s (*p* = 0.006) from baseline to three months post-treatment, 0.24 m/s (*p* = 0.001) from baseline to six months post-treatment, and 0.21 m/s (*p* = 0.001) from baseline to 12 months post-treatment. No statistically significant changes occurred between any of the post-treatment follow-up periods (*p* > 0.999). Due to near significance of the three-month follow-up, we also calculated the Cohen’s d effect sizes which were all 1.0 or greater, indicating a strong effect. Figure 3 shows the mean gait speed, associated *p*-values, and effect sizes at each of the six time frames. Additionally, while the focus of this paper is retention of gait speed, other measures of performance which have been reported (41, 42) showed similar patterns of improvement and retention. Supplementary Figure S1 shows outcome boxplots and association statistics.

### 3.3 Minutes of treatment

The 18 study participants completed an average of 11.7 treatment sessions (range 9–12 sessions) and 290 minutes on the device (range 165–360 minutes) out of a maximum 360 minutes of device treatment. Documented primary reasons for reduced completion included scheduling conflicts and fatigue. A strong, statistically significant relationship was found between treatment duration (measured in minutes of treatment on the gait device) and gait speed at 12 months post-treatment; *r* (16)=0.60, *p* = 0.009.

### 3.4 Minimal clinically important difference

Gait speed changes of the individual participants, mean improvements by time period, and percentages of participants exceeding the gait speed MCID (40) threshold of 0.16 m/s at each time frame are shown in Table 2. Numbers in bold indicate an improvement beyond the MCID value. Participants whose 10MWT included a “turn” due to spatial constraints are identified with an asterisk (*), and participants that received any additional therapy services throughout the entire clinical trial period are identified with a “TX.”

**Table 2:** Individual participant gait speed changes compared to the MCID.

Percentage of time frames change > MCID	ID	Baseline gait speed (m/s)	Gait speed change (m/s) 1Wk post	Gait speed change (m/s) 1Mo post	Gait speed change (m/s) 3Mo post	Gait speed change (m/s) 6Mo post	Gait speed change (m/s) 12Mo post
100%	A	0.20	**+0.44**	**+0.75**	**+0.72**	**+0.56**	**+0.46**
	B	0.21	**+0.27**	**+0.35**	**+0.51**	**+0.29**	**+0.30**
	C*	0.25	**+0.45**	**+0.65**	**+0.49**	**+0.66**	**+0.45**
	D ^TX^	0.30	**+0.16**	**+0.23**	**+0.20**	**+0.34**	**+0.29**
	E*	0.44	**+0.22**	**+0.27**	**+0.23**	**+0.27**	**+0.23**
	F	0.53	**+0.20**	**+0.25**	**+0.28**	**+0.37**	**+0.29**
	G*	0.63	**+0.40**	**+0.41**	**+0.39**	**+0.37**	**+0.38**
80%	H*^TX^	0.61	**+0.56**	**+0.49**	**+0.59**	**+0.30**	+0.07
	I	0.75	**+0.19**	−0.05	**+0.18**	**+0.27**	**+0.41**
	J ^TX^	0.46	+0.13	**+0.17**	**+0.18**	**+0.23**	**+0.27**
60%	K ^TX^	0.78	**+0.61**	**+0.67**	**+0.50**	−0.13	+0.06
	L	0.98	**+0.43**	**+0.32**	−0.17	**+0.29**	+0.06
	M	0.39	**+0.21**	+0.14	+0.01	**+0.21**	**+0.20**
	N*	0.64	+0.14	+0.14	**+0.19**	**+0.22**	**+0.27**
40%	O* ^TX^	0.86	**+0.33**	**+0.22**	−0.15	−0.16	−0.06
20%	P ^TX^	0.39	+0.11	+0.12	**+0.20**	+0.15	+0.07
	Q	0.91	−0.03	−0.09	+0.13	**+0.17**	+0.05
0%	R	0.22	+0.10	+0.03	+0.05	0.00	0.00
	Mean	0.53 (SD 0.25)	**+0.27** **(SD 0.17)**	**+0.28** **(SD 0.24)**	**+0.25** **(SD 0.24)**	**+0.24** **(SD 0.20)**	**+0.21** **(SD 0.16)**
	% of participants >MCID	n/a	72.2%	66.7%	72.2%	77.8%	61.1%

Numbers in bold indicate an improvement beyond the MCID value. m/s, meters per second; Wk, week; Mo, month; Post, post-treatment; MCID, minimal clinically important difference; SD, standard deviation. *, Participants who had a turn during their gait speed assessment. TX, Participants who received some additional therapy during post-treatment follow-ups.

### 3.5 Gait speed classification

**Figure 4:**
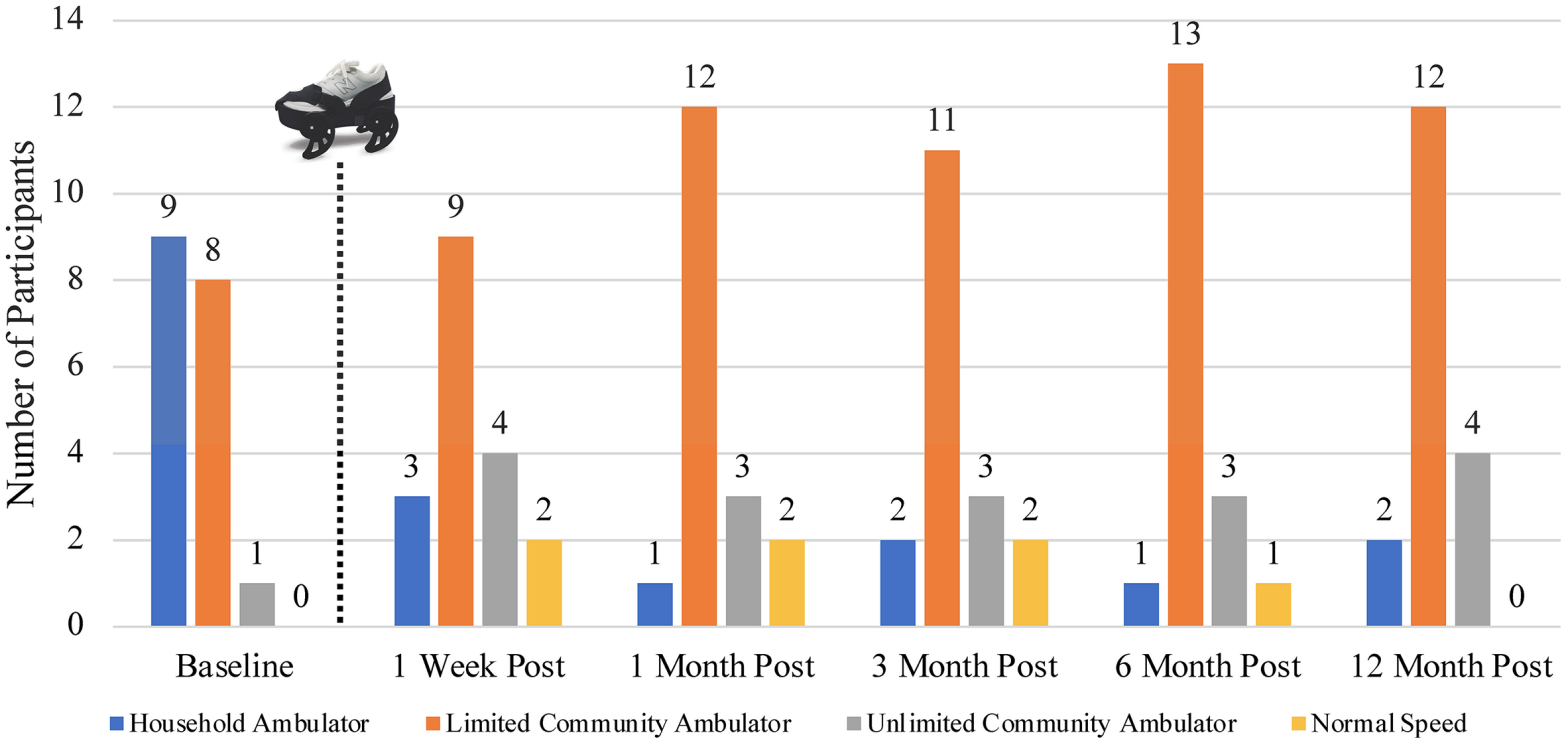
Number of participants in each gait speed category at each study time period utilizing the classifications proposed by Fulk (27). The dotted line and gait device image represent the sequence within the study activities that the device treatment occurred.

Using the Fulk gait speed classifications, at baseline, nine of the participants were classified as household ambulators, eight as limited community ambulators, and one as an unlimited community ambulator (27). After the device treatment, only between one and three participants remained household ambulators throughout the remainder of the study period. The remaining participants in this initial category improved one or two gait speed classifications. Twelve months post-treatment, two participants remained household ambulators, 12 were classified as limited community ambulators, and four improved to unlimited community ambulators. Figure 4 shows the number of participants in each gait speed classification during all study time frames (27). Additionally, in Figure 4 we highlight that one or two participants achieved a gait speed categorized as “normal speed” (at or above 1.2 m/s) during four of the five assessments after treatment with the gait device (23).

### 3.6 Questionnaire responses

The questionnaire consisted of multiple choice questions regarding observations of potential gait speed changes, functional independence changes, and clinical trial experience. (Note: questionnaire responses are only available for 17 of the 18 participants). Fifteen of 17 participants (88%) reported noticing an improvement in their walking speed, 12 of 17 participants (71%) reported improved functional independence, and 17 of 17 (100%) reported a positive clinical trial experience with the gait device. Questions and response percentages are shown in Table 3.

**Table 3:** Questionnaire items and responses.

Survey question	Responses
Do you feel that the iStride® Gait Solution has helped with your walking speed?	Yes: 15/17 (88%)
	No: 0/17 (0%)
	Not really sure: 2/17 (12%)
Since you started treatment on the iStride® Gait Solution, do you feel that you have gained more independence?	Yes: 12/17 (71%)
	No: 5/17 (29%)
Did you have a positive overall experience with the clinical trial?	Yes: 17/17 (100%)
	No: 0/17 (0%)

## 4 Discussion

The objective of this study was to investigate the long-term gait speed changes after treatment with the iStride® gait device in the home environment for individuals with gait impairments from chronic stroke. A review of the participants’ gait speed changes over the study period demonstrates an average gait speed improvement >0.21 m/s across the measured time frames and a gait speed improvement greater than the MCID (compared to baseline) one-year after treatment for over 60% of participants. These results are supported by the vast majority of participants (88%) who reported a subjective gait speed improvement as noted by questionnaire results at study completion.

The combined mechanisms utilized in the gait device provide a novel approach to gait treatment in the population of chronic stroke. While multiple gait treatment techniques and technologies cite the ability to improve the gait speed of stroke survivors, comparison to studies of various overground gait intervention approaches indicates an improvement of this magnitude (which is also retained and fosters an expected improvement in community participation ability) is notable (43). Specifically, a 2009 analysis of seven studies and nearly 400 participants found an average gait speed improvement of 0.07 m/s after traditional, overground gait training (14). DePaul et al. (44) used a motor-learning-science-based overground walking program, which resulted in an average 0.14 m/s gait speed improvement. Park et al. (45) compared the effects of gait training overground versus treadmills and found the largest gait speed improvement, from any of the training conditions, to be 0.1 m/s. The reported gait speed changes from the present study are approximately two to three times greater than those reported by these gait-focused studies and additionally highlight retention, a critical factor not demonstrated in the prior mentioned studies. Further support of the treatment effect is demonstrated by a strong, statistically significant relationship between treatment duration and gait speed 12 months post-treatment.

The gait speed results of this study also exceed those seen in our laboratory-based pilot study (38), suggesting a benefit for the adapted, home-based treatment protocol described in this study. Many studies have emphasized the impact of treatment “context” on motor adaptation and motor learning after stroke. For example, a contextual mismatch of cues is thought to account for the decreased locomotor transfer to overground walking following split-belt treadmill training (46), and Torres-Oviedo and Bastian (47) found that removing vision to eliminate the visual-proprioceptive contextual mismatch specific to treadmills improved the transfer of treadmill adaptation to natural walking. The greater improvements achieved in the present study compared to the pilot, laboratory-based study yield consideration that contextual differences between laboratory and home treatment environments may also influence outcomes after treatment with the gait device. Similarly, potential benefits of adapting and de-adapting (48) the modified gait pattern with natural, overground ambulation in the home environment following each treatment session is a mechanism that could be further explored as possibly contributing to our study’s findings. Moreover, while not yet entirely understood, the value of rehabilitation at home after stroke is further emphasized by the improved outcomes seen with home-centered approaches such as early supported discharge (49) or the effectiveness of the active “control” home environment condition in the LEAPS study (50, 51). Compared to a laboratory environment, performing this gait treatment in the context of where the individual resides (and performs the majority of their gait activities) may have enhanced the transfer and adoption of a modified gait pattern and facilitated the retained gait speed effects seen in the present study.

Continuing, or at least maintaining the functional improvements achieved during rehabilitation is a primary goal of post-stroke management. However, the factors influencing the retention of functional improvements after stroke are not fully understood. In upper extremity literature, studies have shown that gains in arm function after stroke can be maintained or even improved if use is sufficient (52). While seemingly less studied in the context of gait, multiple published studies suggest that critical gait speed thresholds must be reached to achieve increased levels of community participation (26, 27). The results of our study show that a majority (12/18, 67%) of participants achieved a higher gait speed classification immediately following treatment, and 10/17 (59%) maintained a greater gait speed classification at their 12-month follow-up session compared to baseline (27). Given the long-term, sustained gait speed improvement, these results suggest that an improved walking ability was achieved following treatment that could be maintained through increased participation in regular, daily activities. For example, Partcipant A was interviewed following his treatment with the gait device and described the ability to become substantially more active after treatment with the gait device, including resuming recreational activities. We suspect increased activity levels, such as these, may reinforce and sustain the immediate post-treatment gait speed improvements.

Interestingly, the group of participants that maintained a greater gait speed classification at their 12-month follow-up session includes five of the six participants that were two-years or less post-stroke and the most chronic participant who was 25+ years post-stroke at baseline. These results highlight the value of treatment in the immediate two-year post-stroke time frame and further emphasize that meaningful improvement is achievable even many years post-stroke. Moreover, reviewing the participants’ gait speeds over the 12-month period appears to reveal several unique patterns of gait speed change and retention. Some participants improved their gait speed post-treatment and maintained this improvement through the 12-month follow-up, such as Participants A and C, for example. Others demonstrated initial improvement post-treatment but returned to their original gait speed (approximately) by the 12-month follow-up (such as Participant H), and yet others continued to improve their gait speed over the 12-month period, despite no further treatment (such as Participant I). Future studies would be useful to differentiate these individual trends, as well as the specific characteristics that may have influenced treatment responsiveness and retention.

### 4.1 Limitations

There are several limitations to our study. When possible, consistent physical therapists were used with each of the participants in this study. This consistency, while minimizing interrater reliability issues, does not permit blinding of the therapists. Repeated outcomes testing could also introduce a practice effect, and retrospective recall could limit the accuracy of questionnaire results. Additionally, a lack of a control group limits direct comparison to standard treatments and does not explore the effect of the device in comparison to the concomitant walking activity. However, while the lack of a control group is a limitation, the effect of simply walking on the outcome of gait speed has been thoroughly investigated in the literature (with varying durations, contexts, and intensities explored) (45, 53–55). The outcomes of these studies demonstrate a substantially lesser gait speed effect than noted in the present study. Moreover, it is unlikely that the gait speed results could be attributed to spontaneous improvement given the chronicity of our study population.

As noted in our inclusion criteria, the clinical trial participants did not receive any additional physical therapy treatment from the clinical trial physical therapists or external physical therapists during the treatment period through the one-week follow-up. However, given the extended duration of the study, we did not preclude participants from obtaining services after this time. Six of 18 participants did resume some form of additional physical therapy treatment between the one-week follow-up and 12-month follow-up, as annotated in Table 2. Of these six participants, three received six or fewer total additional therapy sessions. Of the remaining three participants, two received services focusing on upper extremity function (with some full-body therapy included). It is important to note that two-thirds of our participants (12 out of 18 participants) did not receive any additional physical therapy treatment throughout the study duration.

We also encountered challenges related to gait speed assessment in the home environment. While the home environments of the majority of participants (12 of 18) were able to accommodate the spatial requirements for a 10MWT, six of the 18 participants required a “turn” to achieve the full 10 meters of walking due to spatial constraints within the home (and no suitable outdoor alternative). Of these six participants, two performed a turn after six meters, two turned after seven meters, and two turned after nine meters. Challenges as these are commonly encountered during testing in home environments (28), but are likely outweighed by the benefits of capturing outcomes in natural conditions. These participants have been identified with an asterisk in Table 2. Importantly, the overall trends seen in our results with all participants do not change if we remove the participants that had some additional physical therapy (between the one-week and 12-month follow-ups) or those that had a “turn” during their gait evaluation or both of these groups (see Supplementary Tables S1–S3).

## 5 Conclusion

The present study supports the usage of a four-week gait device treatment protocol for chronic stroke survivors in their home environment. Results indicate that the described treatment has the potential to result in long-term, meaningful gait speed improvement for individuals with hemiparetic gait impairments. This treatment appears impactful in its ability to facilitate clinically significant gait speed changes, which have been correlated in the literature with decreased disability and improved quality of life, in a relatively short time frame and from the home environment. Additionally, our findings provide additional support to the notion that novel treatments can enhance recovery, even many years post-stroke. These promising results warrant further study to elucidate the full impact of this home-use gait treatment device.

## Data availability statement

The original contributions presented in the study are included in the article/Supplementary material, further inquiries can be directed to the corresponding author.

## Ethics statement

The studies involving humans were approved by Western Institutional Review Board (now WCG). The studies were conducted in accordance with the local legislation and institutional requirements. The participants provided their written informed consent to participate in this study.

## Author contributions

LR, DH, and KR designed the study. LR screened the participants and obtained informed consent. LR oversaw the study protocol. BD, DH, KR, NT, and SB analyzed the data. BD wrote the manuscript with critical feedback from all authors. All authors contributed to the article and approved the submitted version.
