# Supplementary material for: One-year retention of gait speed improvement in stroke survivors after treatment with a wearable home-use gait device
Source: Front Neurol. 2024 Jan 11;14:1089083. doi: 10.3389/fneur.2023.1089083 (PMC10808505; doi:10.3389/fneur.2023.1089083)
Supplement: Supplementary file 1 [file Table_1.docx]

# Supplementary Table 1. Individual participant gait speed changes compared to the MCID with participants that received some additional therapy during post-treatment follow-ups removed from analysis

| ID | Baseline Gait Speed (m/s) | Gait Speed Change (m/s) 1Wk Post | Gait Speed Change (m/s) 1Mo Post | Gait Speed Change (m/s) 3Mo Post | Gait Speed Change (m/s) 6Mo Post | Gait Speed Change (m/s) 12Mo Post |
| --- | --- | --- | --- | --- | --- | --- |
| A | 0.20 | **+0.44** | **+0.75** | **+0.72** | **+0.56** | **+0.46** |
| B | 0.21 | **+0.27** | **+0.35** | **+0.51** | **+0.29** | **+0.30** |
| C* | 0.25 | **+0.45** | **+0.65** | **+0.49** | **+0.66** | **+0.45** |
| E* | 0.44 | **+0.22** | **+0.27** | **+0.23** | **+0.27** | **+0.23** |
| F | 0.53 | **+0.20** | **+0.25** | **+0.28** | **+0.37** | **+0.29** |
| G* | 0.63 | **+0.40** | **+0.41** | **+0.39** | **+0.37** | **+0.38** |
| I | 0.75 | **+0.19** | -0.05 | **+0.18** | **+0.27** | **+0.41** |
| L | 0.98 | **+0.43** | **+0.32** | -0.17 | **+0.29** | +0.06 |
| M | 0.39 | **+0.21** | +0.14 | +0.01 | **+0.21** | **+0.20** |
| N* | 0.64 | +0.14 | +0.14 | **+0.19** | **+0.22** | **+0.27** |
| Q | 0.91 | -0.03 | -0.09 | +0.13 | **+0.17** | +0.05 |
| R | 0.22 | +0.10 | +0.03 | +0.05 | 0.00 | 0.00 |
| Mean | 0.51 | **+0.25** | **+0.26** | **+0.25** | **+0.31** | **+0.26** |
| % of Participants >MCID | n/a | 66.7% | 72.2% | 66.7% | 94.4% | 83.3% |

Numbers in bold indicate an improvement beyond the MCID value. m/s, meters per second; Wk, week; Mo, month; Post, post-treatment; MCID, minimal clinically important difference; *, participants who had a turn during their gait speed assessment.
