# Supplementary material for: One-year retention of gait speed improvement in stroke survivors after treatment with a wearable home-use gait device
Source: Front Neurol. 2024 Jan 11;14:1089083. doi: 10.3389/fneur.2023.1089083 (PMC10808505; doi:10.3389/fneur.2023.1089083)
Supplement: Supplementary file 2 [file Table_2.docx]

# Supplementary Table 2. Individual participant gait speed changes compared to the MCID with participants that had a “turn” during the 10MWT removed from analysis

| ID | Baseline Gait Speed (m/s) | Gait Speed Change (m/s) 1Wk Post | Gait Speed Change (m/s) 1Mo Post | Gait Speed Change (m/s) 3Mo Post | Gait Speed Change (m/s) 6Mo Post | Gait Speed Change (m/s) 12Mo Post |
| --- | --- | --- | --- | --- | --- | --- |
| A | 0.20 | **+0.44** | **+0.75** | **+0.72** | **+0.56** | **+0.46** |
| B | 0.21 | **+0.27** | **+0.35** | **+0.51** | **+0.29** | **+0.30** |
| D ^TX^ | 0.30 | **+0.16** | **+0.23** | **+0.20** | **+0.34** | **+0.29** |
| F | 0.53 | **+0.20** | **+0.25** | **+0.28** | **+0.37** | **+0.29** |
| I | 0.75 | **+0.19** | -0.05 | **+0.18** | **+0.27** | **+0.41** |
| J ^TX^ | 0.46 | +0.13 | **+0.17** | **+0.18** | **+0.23** | **+0.27** |
| K ^TX^ | 0.78 | **+0.61** | **+0.67** | **+0.50** | -0.13 | +0.06 |
| L | 0.98 | **+0.43** | **+0.32** | -0.17 | **+0.29** | +0.06 |
| M | 0.39 | **+0.21** | +0.14 | +0.01 | **+0.21** | **+0.20** |
| P ^TX^ | 0.39 | +0.11 | +0.12 | **+0.20** | +0.15 | +0.07 |
| Q | 0.91 | -0.03 | -0.09 | +0.13 | **+0.17** | +0.05 |
| R | 0.22 | +0.10 | +0.03 | +0.05 | 0.00 | 0.00 |
| Mean | 0.51 | **+0.24** | **+0.24** | **+0.23** | **+0.23** | **+0.21** |
| % of Participants >MCID | n/a | 66.7% | 58.3% | 66.7% | 75.0% | 58.3% |

Numbers in bold indicate an improvement beyond the MCID value. m/s, meters per second; Wk, week; Mo, month; Post, post-treatment; MCID, minimal clinically important difference; TX, participants who received some additional therapy during post-treatment follow-ups.
