# Supplementary material for: One-year retention of gait speed improvement in stroke survivors after treatment with a wearable home-use gait device
Source: Front Neurol. 2024 Jan 11;14:1089083. doi: 10.3389/fneur.2023.1089083 (PMC10808505; doi:10.3389/fneur.2023.1089083)
Supplement: Supplementary file 4 [file Data_Sheet_1.docx]

# Supplementary Figure 1. Distribution of Gait Speed, Timed Up and Go Test, and Berg Balance Scale scores at baseline and five post-treatment time periods.


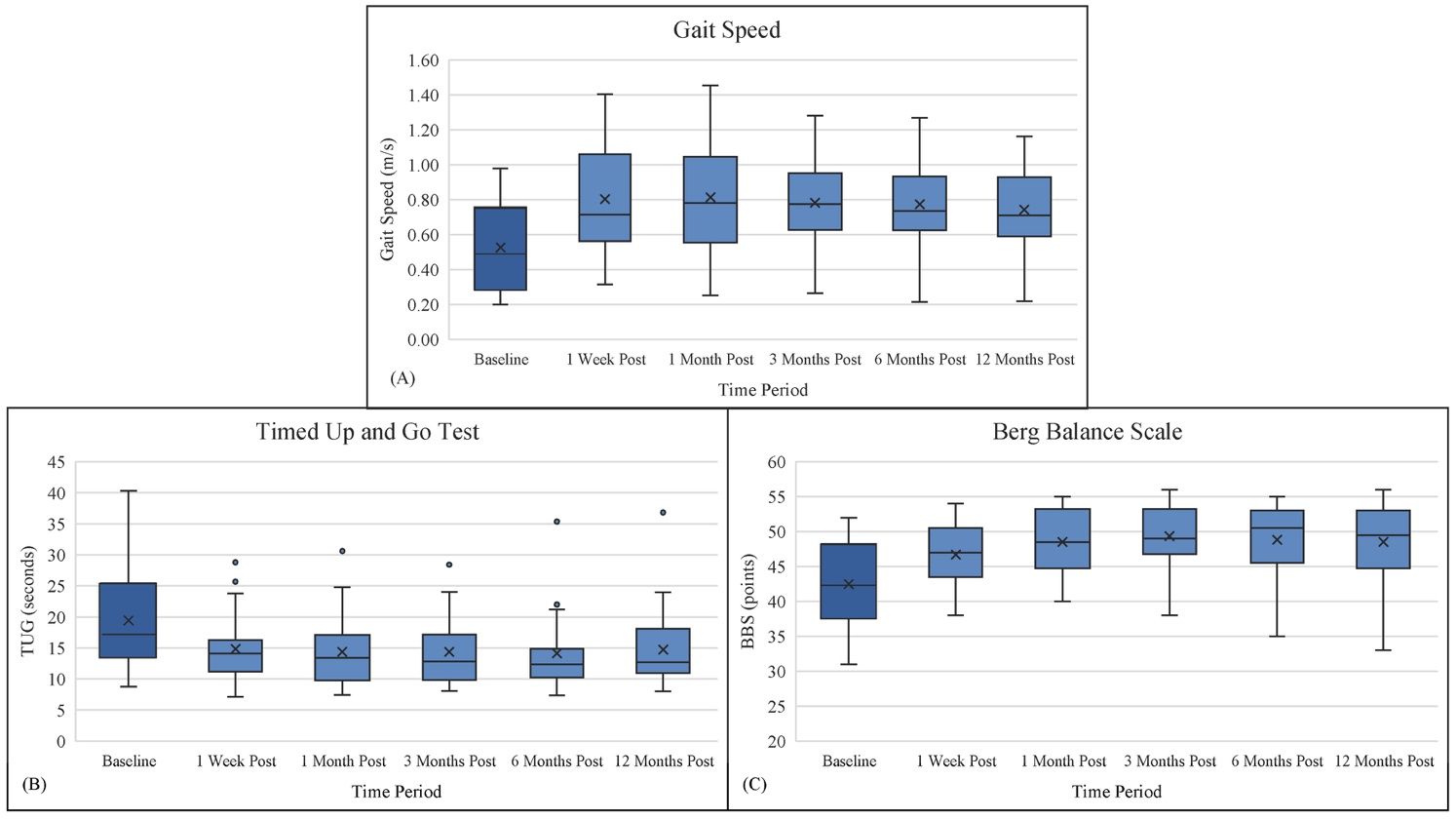


**Supplementary Figure 1.** Box plots of three outcome measures at baseline and five post-treatment time periods: 1(A) Gait Speed, 1(B) Timed Up and Go Test (TUG), and 1(C) Berg Balance Scale (BBS). Horizontal lines represent median values and x symbols represent means. Score distributions across the three outcomes show a similar pattern of score improvement and retention after treatment with the gait device. (Improvement results in higher gait speeds and BBS scores and lower TUG scores.) Strong, statistically significant correlations were found between the balance-focused variables (TUG and BBS) and gait speed. Pearson’s correlation coefficient was calculated to assess the relationship between gait speed and TUG scores 12 months post-treatment. There was a strong, negative relationship between these variables; r(16)=-.84, p<0.001. Spearman rank correlation was calculated to assess the relationship between gait speed and BBS. There was a moderately strong, positive association between these variables; r_s_(16)=.52, p=0.03.
